# Lectin RCA-I specifically binds to metastasis-associated cell surface glycans in triple-negative breast cancer

**DOI:** 10.1186/s13058-015-0544-9

**Published:** 2015-03-11

**Authors:** Shu-Min Zhou, Li Cheng, Shu-Juan Guo, Yang Wang, Daniel M Czajkowsky, Huafang Gao, Xiao-Fang Hu, Sheng-Ce Tao

**Affiliations:** Shanghai Center for Systems Biomedicine, Key Laboratory of Systems Biomedicine (Ministry of Education), Shanghai Jiao Tong University, 800 Dongchuan Road, Shanghai, 200240 China; State Key Laboratory of Oncogenes and Related Genes, Shanghai Jiao Tong University, 800 Dongchuan Road, Shanghai, 200240 China; Bio-ID Center, School of Biomedical Engineering, Shanghai Jiao Tong University, 800 Dongchuan Road, Shanghai, 200240 China; Institute for Microsurgery of Limbs, Shanghai Sixth hospital, Shanghai Jiao Tong University, 800 Dongchuan Road, Shanghai, 200240 China; Human Genetic Resource Center, National Research Institute for Family Planning, 12 Dahuisi Road, Beijing, 100081 China

## Abstract

**Introduction:**

Triple-negative breast cancer (TNBC) patients often face a high risk of early relapse characterized by extensive metastasis. Previous works have shown that aberrant cell surface glycosylation is associated with cancer metastasis, suggesting that altered glycosylations might serve as diagnostic signatures of metastatic potential. To address this question, we took TNBC as an example and analyzed six TNBC cell lines, derived from a common progenitor, that differ in metastatic potential.

**Methods:**

We used a microarray with 91 lectins to screen for altered lectin bindings to the six TNBC cell lines. Candidate lectins were then verified by lectin-based flow cytometry and immunofluorescent staining assays using both TNBC/non-TNBC cancer cells. Patient-derived tissue microarrays were then employed to analyze whether the staining of *Ricinus communis* agglutinin I (RCA-I), correlated with TNBC severity. We also carried out real-time cell motility assays in the presence of RCA-I. Finally, liquid chromatography-mass spectrometry/tandem spectrometry (LC-MS/MS) was employed to identify the membrane glycoproteins recognized by RCA-I.

**Results:**

Using the lectin microarray, we found that the bindings of RCA-I to TNBC cells are proportional to their metastatic capacity. Tissue microarray experiments showed that the intensity of RCA-I staining is positively correlated with the TNM grades. The real-time cell motility assays clearly demonstrated RCA-I inhibition of adhesion, migration, and invasion of TNBC cells of high metastatic capacity. Additionally, a membrane glycoprotein, POTE ankyrin domain family member F (POTEF), with different galactosylation extents in high/low metastatic TNBC cells was identified by LC-MS/MS as a binder of RCA-I.

**Conclusions:**

We discovered RCA-I, which bound to TNBC cells to a degree that is proportional to their metastatic capacities, and found that this binding inhibits the cell invasion, migration, and adhesion, and identified a membrane protein, POTEF, which may play a key role in mediating these effects. These results thus indicate that RCA-I-specific cell surface glycoproteins may play a critical role in TNBC metastasis and that the extent of RCA-I cell binding could be used in diagnosis to predict the likelihood of developing metastases in TNBC patients.

**Electronic supplementary material:**

The online version of this article (doi:10.1186/s13058-015-0544-9) contains supplementary material, which is available to authorized users.

## Introduction

Breast cancer is the leading cause of death from cancer in women and indeed one of the most prevalent types of cancers worldwide [1]. Triple-negative breast cancer (TNBC) accounts for 15 to 20% of all breast cancers and is associated with the worst prognosis [2]. TNBC is characterized by a lack of estrogen receptor/progesterone receptor (ER/PgR) expression and an absence of human epidermal growth factor receptor 2 (HER2) overexpression or amplification, which makes it insensitive to hormone or trastuzumab treatment [3]. As such, the most common current treatment option for TNBC patients is cytotoxic chemotherapy [4]. Yet with this, even for patients who at first respond well to therapy, there is a high rate of early relapse [5] and a poor long-term outcome [6]. Moreover, there is presently no effective treatment for TNBC patients with many metastatic niches [7]. Hence, with such a poor prognosis and tendency to relapse with distant metastases, there is an urgent medical need to understand the mechanisms underlying metastasis in TNBC to develop better therapy and methods of early diagnosis.

Recently, many studies have found that the occurrence or progression of a number of different tumors is associated with aberrant protein glycosylation. For example, unusual sialylation and fucosylation [8], increased branching of *N*-glycans [9], and truncated *O*-glycans [10] have all been observed in tumors and correlated with tumor development. Altered glycans on cell surfaces have also recently been implicated in the development of metastases [11]. For example, core-3-derived glycans were reported to be downregulated in metastatic pancreatic cancer [12] and a global decrease in larger branched tri-and tetra-antennary *N*-linked glycans was found to be correlated with the progression of prostate cancer [13]. Yet a thorough understanding of the changes in cell surface glycans during the progression of cancer requires techniques capable of profiling and analyzing an entire cell surface repertoire of glycans. Lectin (sugar-binding protein) microarrays have recently emerged as a significantly promising technology for obtaining such whole-cell glycan characterizations in a very rapid fashion [14,15]. With nearly 100 different arrayed lectins, such highly multiplexed characterization enables the possibility for the detection of fine differences between cells and the identification of specific lectins responsible for these differences, which may then be explored in diagnostic applications [14].

In the present study, a high-density lectin microarray containing 91 lectins was used to compare the cell surface glycan patterns of a TNBC cell line (MDA-MB-231) and five other cell lines of different metastatic potential that are derived from this cell line [16]. Surprisingly, we found that a single terminal galactose-specific lectin, *Ricinus communis* agglutinin I (RCA-I), binds to these cells to a degree that is proportional to their metastatic capacity. This result was confirmed in RCA-I binding experiments using TNBC patient-derived tissue microarrays, where greater binding was observed to later-stage tumors with high metastatic capacity [17]. By comparison, there was no correlation between the extent of RCA-I binding and the clinical stage of non-TNBC tissue. Moreover, somewhat unexpectedly, we also found that RCA-I specifically blocked the adhesion, invasion, and migration of the cell lines with greater metastatic potential. In addition, using LC-MS/MS and stable isotope labeling by amino acids in cell culture (SILAC), we identified a membrane glycoprotein, POTE ankyrin domain family member F (POTEF), showing different extents of galactosylation in high versus low metastatic TNBC cells. Overall, these results point to a role of RCA-I-specific membrane glycans in TNBC metastasis and, importantly, identify RCA-I as a potential diagnostic or therapeutic agent of this presently poorly treated cancer.

## Methods

### Chemicals and reagents

All of the lectins were purchased from EY Laboratories (San Mateo, CA, USA) or Vector Laboratories (Burlingame, CA, USA) unless otherwise indicated. All the cell culture media and serum were from Life Technologies (Carlsbad, CA, USA) unless otherwise indicated. Carboxyfluorescein diacetate succinimidyl ester (CFDA-SE) was also from Life Technologies (Carlsbad, CA, USA). Cy3-streptavidin and paraformaldehyde (PFA) were from Sigma-Aldrich China (Shanghai, China). DAPI was from Roche Diagnostics (Mannheim, Germany).

### Cell cultures

The MBA-MD-231 cell line and five other descendant cell lines (SCP2, SCP4, SCP6, SCP28 and 4175) were kindly provided by Professor Yinbin Kang of Princeton University. Other breast cancer lines (MCF7, SKBR3, BT549 and SUM159) were from American Type Culture Collection (ATCC, Manassas, VA, USA) or China Center for Type Culture Collection, (CCTCC, Wuhan University, Wuhan, China). MBA-MD-231, SCP2, SCP4, SCP6, SCP28, 4175 and MCF7 were cultured in Dulbecco’s modified Eagle’s medium (DMEM) with 10% fetal bovine serum (FBS); BT549 was cultured in PRIM01640 with 10% FBS; SKBR3 was cultured in McCoy’s 5A with 10% FBS; SUM159 was cultured in F12K with 10% FBS. All cell lines were passaged every 2 to 3 days when the cell confluence reached about 80%.

### Lectin microarray screening for surface glycans on live cells

The fabrication of the lectin microarray and profiling of live cells was performed as previously described [14]. Briefly, the cells were harvested by trypsin digestion and fluorescently labeled with CFDA-SE. The cells were then resuspended in binding buffer (phosphate-buffered saline (PBS) with 0.5 mM CaCl_2_, 0.1 mM MnCl_2_ and 1% bovine serum albumin (BSA)) and 5 × 10^5^ cells were probed per block on the lectin microarray. After incubation for 1 hr at room temperature, the microarray was washed in PBST (PBS with 0.5% Tween-20). The bound cells were detected by a GenePix 4200A scanner and the fluorescence intensity of each spot was measured with GenePix Pro 6.0 Software (Molecular Devices, Sunnyvale, CA, USA). Lectins that displayed signal intensities of greater than or equal to three standard deviations above background were defined as positive signals.

### Flow cytometry (FCM) analysis of lectin-cell binding

We followed a protocol slightly modified from that previously described [18]. Briefly, 1 × 10^5^ cells of each cell line were resuspended in serum-free medium and incubated with 50 μg/ml of biotinylated lectin for 15 min at 18°C. The cells were then washed three times with PBS and incubated with Cy3-streptavidin at 1 μg/ml. After three washes, cells were resuspended in FACS Flow (BD Biosciences, Franklin Lakes, NJ, USA) supplemented with 0.1% BSA and analyzed in a FacsCalibur™ flow cytometer (BD Biosciences, Franklin Lakes, NJ, USA) using CellQuest software (BD Biosciences, Franklin Lakes, NJ, USA), following a standard protocol.

### Lectin staining assay

This assay was carried out as described [19]. Briefly, cells cultured in 24-well plates were fixed with 4% PFA for 30 min on ice. The fixed cells were then incubated with biotinylated lectin at 50 μg/ml for 1 hr at room temperature, followed by incubation with Cy3-conjugated streptavidin. DAPI staining was also carried out on the same sample. The results were analyzed by microscopy (DMI6000B, Leica, Jena, Germany).

### Transwell assays for cell invasion

This assay was performed as previously described [20] with slight modification. Briefly, cells were starved in serum-free medium for 20 hr. Single-cell suspensions were then prepared in serum-free medium and incubated with RCA-I at concentrations of 0, 1, 2.5 and 5 μg/ml. The pre-blocked cells were resuspended and added to the upper chamber of matrigel-coated inserts with an 8-μm pore polycarbonate membrane in a 24-well transwell plate (Corning Costar Corp., Cambridge, MA, USA). The cells were allowed to invade toward medium containing 10% FBS in the lower chambers. After 20 hr incubation, cells on the upper surface of the membrane were wiped off with a cotton swab, and the cells that had migrated below the membrane were stained with CFDA-SE, and examined using fluorescence microscopy.

### Real-time cell analyzer (RTCA) assays for cell adhesion, migration and invasion

Experiments were carried out using the xCELLigence RTCA DP (Roche Diagnostics GmbH, Mannheim, Germany) as described [21]. For the migration and invasion assays, a CIM-16 plate (Roche Diagnostics GmbH, Mannheim, Germany) was used with (for invasion) or without (for migration) matrigel coating. The cells were first incubated with RCA-I at various concentrations and resuspended in 100 μl serum-free medium. The cells (4 × 10^4^ per well) were then seeded into each well of the upper chamber of the CIM-16 plate. For the invasion assay, the cells were incubated for about 24 hr in a cell incubator and the cell index (CI) was monitored every 5 min. The CI is linearly related to the number of cells [21]. Four replicates of each cell concentration were used in each test. For the migration experiments, the whole process was similar, but the incubation time was much shorter (approximately 12 hr). For the adhesion assay, an E-plate 16-well plate (Roche Diagnostics GmbH, Mannheim, Germany) was used and the whole process was similar to that for the analysis of cell proliferation [22]. The CI was monitored every 1 min for about 1 hr. Four replicates of each cell concentration were carried out in each test.

### Tissue microarray analysis

The TNBC tissue microarray containing 52 TNBC clinic samples of different TNM grades and breast cancer tissue microarray of all types with survival time containing 160 clinic samples were purchased from Shanghai Outdo Biotech Company (Shanghai, China). Shanghai Outdo Biotech Company is a daughter company of Shanghai Biochip Co., Ltd., which is also the National Engineering Center for Biochip Design and Engineering in Shanghai. The tissue samples on the tissue microarrays that we used in this study were collected from Tai Zhou hospital of Zhejiang province, China. All the patients had been given informed consent and the collection of tissue samples for research was approved by the ethics committee of Tai Zhou hospital. The immunohistochemistry (IHC) assay using RCA-I was performed as described [23]. Briefly, the slides were first deparaffinized, followed by blocking with 30% normal donkey serum. Biotinylated lectin was then added onto the slide (50 μg/ml) and incubated for 1 hr, followed by incubation with horseradish peroxidase (HRP)-conjugated streptavidin and the signal was visualized by using a DAB protein kit (Sigma-Aldrich China Inc., Shanghai, China). The specimens were analyzed under a light microscope (Nikon, Tokyo, Japan) by pathologists.

The extent (%) of RCA-I binding was calculated as the ratio of positive cells to total cancer cells, and a value of 10% or higher was defined as a positive signal [2]. In addition, RCA-I staining intensity was defined from 0 to 4 based on the color shades of IHC staining. Statistical analysis was performed by GraphPad Prism 5.0 (GraphPad Software Inc., San Diego, CA, USA).

### SILAC labeling and membrane protein extraction

TNBC cell lines SCP6 were cultured in DMEM containing heavy ^13^C-labeled lysine supplemented with dialyzed FBS (Pierce, Rockford, IL, USA) while 4175 cells were cultured in DMEM containing light (^12^C) lysine for a minimum of six population doublings as described in reference [24]. Membrane protein extraction was performed using the Mem-PER Kit (Pierce, Rockford, IL, USA) with protease cocktail inhibitors (Roche Diagnostics GmbH, Mannheim, Germany) according to standard procedures.

### Capture of membrane glycoproteins by RCA-I affinity

Biotinylated RCA-I was immobilized onto streptavidin magnetic beads (Invitrogen, Carlsbad, CA, USA) as described in reference [25]. Glycoprotein capture was performed as in reference [26]. Briefly, cell lysate containing 800 μg protein extracted from ^13^C-labeled SCP6 cells and ^12^C-labeled 4175 cells each were mixed at a 1:1 ratio and diluted four times with binding buffer (20 mM Tris-HCl, pH7.4, 150 mM NaCl, 1 mM MgCl_2_, 1 mM CaCl_2_, and 1 mM MnCl_2_). The lysate mixture was then incubated with RCA-I-coated magnetic beads for 2 hr at 4°C to capture the membrane glycoproteins and the captured glycoproteins were released with 100 μL elution buffer (200 mM D-galactose in binding buffer, pH 7.4).

### Liquid chromatography-mass spectrometry/tandem spectrometry (LC-MS/MS) and data analysis

For LC-MS/MS, the assay was performed as described in reference [26] with modifications. In brief, about 1.5 mg of total protein was separated by 12% SDS-PAGE gel and was cut into bands and then diced into 1 cm^3^ cubes. In-gel trypsin digestion was done according to standard procedures [27]. Peptide samples were then analyzed using a high-throughput electrospray ion-trap mass spectrometer amaZon ETD (Bruker Daltonics, Bremen, Germany) combined with an Ultimate™ 3000 Nano-LC system (Dionex, Sunnyvale, CA, USA). Mass spectra were recorded in the stand-enhanced mode at a speed of 8,100 m/z. Tandem mass spectra were acquired in the ultra-scan operating mode at 26,000 m/z/s (Bruker Esquire Control software, Bruker Daltonics, Bremen, Germany). The raw files were processed using the LC/MS software DataAnalysis 4.0 (Bruker Compass software, Bruker Daltonics, Bremen, Germany) and converted into mgf files for MASCOT 2.3 (Matrix Science, Boston, MA, USA) evaluation using a forward and reverse human protein database from International Protein Index (IPI).

## Results

### Metastasis-specific lectin binding identified on TNBC cell lines

**Figure 1:**
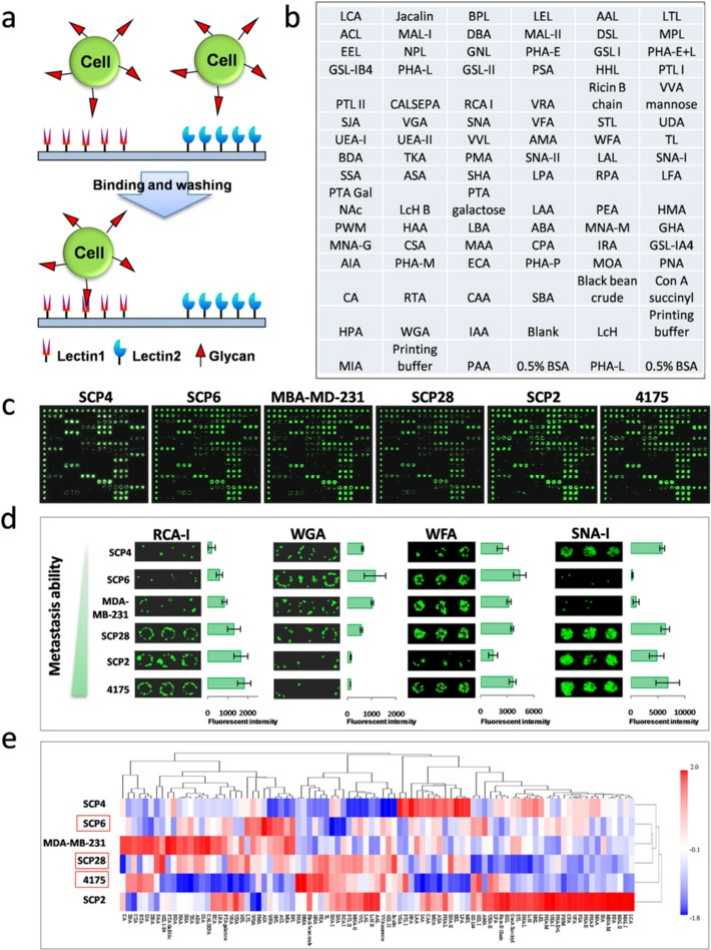
**Identification of metastasis-specific lectin binding to TNBC cells. (a)** Schematic of the probing of fluorescently labeled cells on the lectin microarray. **(b)** Layout of the lectin microarray containing 91 lectins. **(c)** Representative lectin microarray binding patterns of the six TNBC cell lines. **(d)** Four lectins exhibited different extents of binding to the six different TNBC cell lines. The indicated intensities are represented as the mean values ± standard deviation (SD), *n* = 4. **(e)** Clustered heat map of the lectin cell-binding profiles. This map was generated and clustered according to the lectin-binding pattern and mean intensity. The order of the cells in this map closely matches their order of metastatic capacity. TNBC, triple-negative breast cancer.

Six TNBC cell lines (SCP4, SCP6, MDA-MB-231, SCP28, SCP2 and 4175) that differ in metastatic capacity were chosen in this study owing to their common origin and extensive previous characterization [28,29]. To identify the cell surface glycan profiles associated with each of these cell lines, freshly harvested cells were first stained by CFDA-SE and then incubated on a lectin microarray containing 91 lectins, in which each lectin was present in triplicate [14] (Figure 1a-c). Overall, we found that these cells bound between 43 and 49 lectins (Figure S1 in Additional file 1). Of these, 41 were common among all cell lines, and, of the remaining, six were common among at least two cell lines. This high degree of overlap is probably owing to the fact that these cells were derived from a common progenitor. Among the lectins that were observed to bind to these cell lines, four (namely, RCA-I, *Triticum vulgaris* agglutinin (WGA), *Wisteria floribunda* agglutinin (WFA) and *Sambucus nigra* agglutinin I (SNA-I)) were found to show noticeably different extents of binding to different cell lines (Figure 1d). Of note, the binding of RCA-I (a protein found in the plant, *Ricinus communis*) to these cell lines was proportional to their metastatic capacity, while that of WGA was inversely proportional. Surprisingly, SNA-I, which was previously reported to show an enhanced binding to highly metastatic cancer cells [30] did not exhibit stronger binding to the TNBC cells of greater metastatic potential, indicating that the sialic acid content of the surface glycans may not correlate with the metastatic capacity of these cells. To better compare the overall lectin-binding profiles of these cells, a heat map was generated and clustered according to the lectin-binding pattern and intensity (Figure 1e). Clear differences in lectin-binding patterns, and thus of the accessible cell surface glycans, were present between the six cell lines. Interestingly, the order of the TNBC cell lines in this cluster is the same as the order of their known metastatic capacity, except for the switch in order of the two cell lines with the highest metastatic potential (4175 and SCP2). This finding was also observed if the binding patterns of RCA-I and WGA were excluded from the heat map (data not shown).

### Validation of metastasis-specific lectin-binding patterns in TNBC and non-TNBC cells

**Figure 2:**
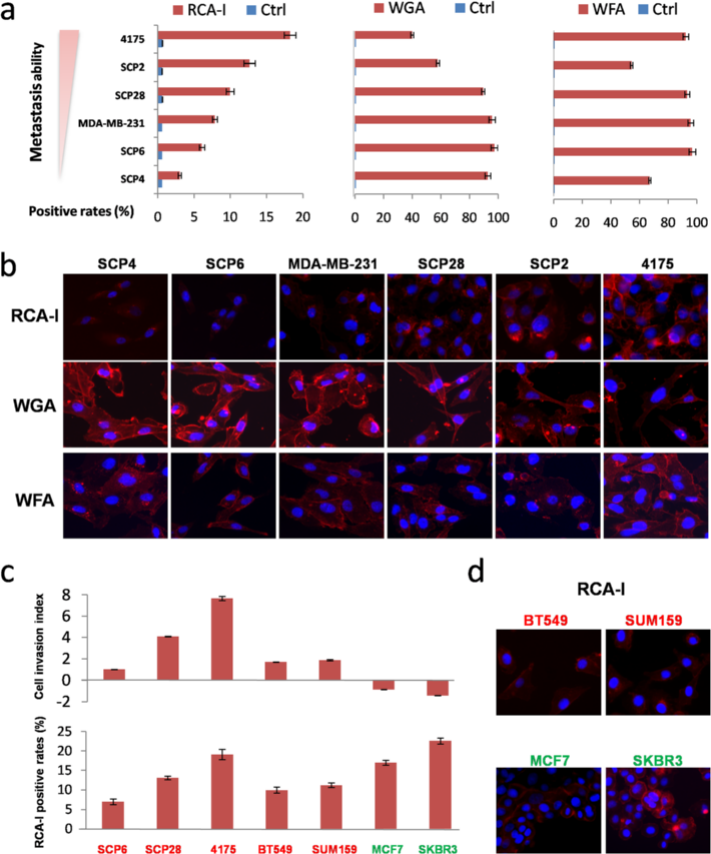
**The RCA-I-binding tendency is constant with the invasion ability of TNBC cells rather than those of non-TNBC cells. (a)** FCM analysis confirms the proportional, inversely proportional, and uncorrelated binding to the metastatic capacity of these cells with RCA-I, WGA, and WFA, respectively. **(b)** Incubation of biotinylated RCA-I, WFA, or WGA, followed by the addition of Cy3-conjugated streptavidin and direct inspection with microscopy further confirmed the cell-binding tendencies of these lectins. **(c)** The RCA-I-binding positive rates of five TNBC (red) and two non-TNBC cells (green) were analyzed by FCM, which were referred to their relative metastatic abilities. **(d)** The immunofluorescent staining of RCA-I binding to another two TNBC cells (SUM159 and BT549) and two non-TNBC cells (MCF7 and SKBR3). FCM, flow cytometry; RCA-I, *Ricinus communis* agglutinin I; TNBC, triple-negative breast cancer; WFA, *Wisteria floribunda* agglutinin; WGA, *Triticum vulgaris* agglutinin.

To verify the results from the lectin microarray, representative interactions were evaluated by lectin-based flow cytometry, using biotinylated lectins and Cy3-labeled streptavidin for detection. The lectins investigated were representative of three categories: namely, those that bound proportionally (RCA-I), inversely proportionally (WGA), or whose binding was uncorrelated with metastatic capacity (WFA). As summarized in Figure 2a, and Figure S2a in Additional file 2, we found that the FCM data indeed exhibited the same binding tendencies as that of the lectin microarray. To further confirm this result in more TNBC cells and analyze whether RCA-I binding exhibited a similar tendency in non-TNBC cancer cells, an additional four breast cancer cell lines (two TNBCs and two non-TNBCs) were also evaluated with RCA-I-based FCM. As shown in Figure 2c, RCA-I indeed bound strongly to these other TNBC cells. Interestingly, RCA-I also exhibited extensive binding to the non-TNBC cancer cells.

We also sought further validation of these results by direct visualization of cells incubated with these biotinylated lectins followed by Cy3-streptavidin. As shown in Figure 2b and d, the binding properties from the microarray experiments and RCA-I-based FCM assays were also observed by direct inspection of these labeled cells.

### Binding of RCA-I to tissues from TNBC patients

**Figure 3:**
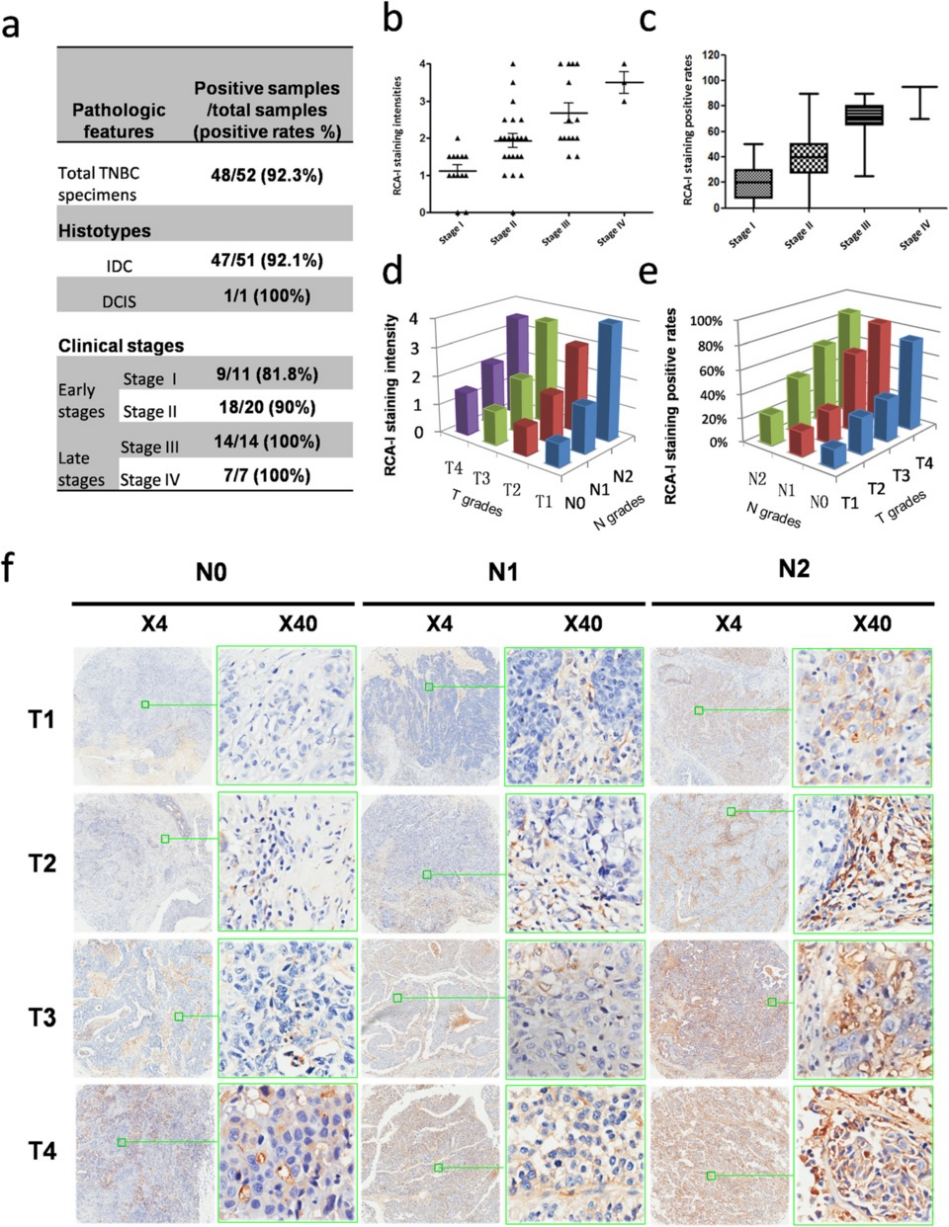
**Evaluation of RCA-I binding to clinical TNBC samples in a tissue microarray. (a)** Overall, a very high proportion of these samples was bound by RCA-I. There was also a dependence of the rate of positive RCA-I labeling on the clinical stage of the tissue, with later-stage tissues exhibiting a higher binding rate than early-stage tissues. **(b, c)** The dependence of the mean intensities and positive rates of RCA-I membrane staining on the tumor stages. **(d, e)** The dependence of the mean intensities and positive rates of RCA-I staining on the T and N grades. **(f)** Representative views of TNBC tissues of different TNM grades that were incubated with biotinylated RCA-I and Cy3-conjugated streptavidin. RCA-I, *Ricinus communis* agglutinin I; TNBC, triple-negative breast cancer.

In order to examine the potential clinical relevance of the observed relationship between membrane RCA-I binding and the metastatic capacity of the TNBC cells, we investigated the extent to which RCA-I binds to breast cancer tissues of TNBC patients. In particular, we employed a tissue microarray containing 52 clinical samples from TNBC patients (median age 52.7 years, range 34 to 79 years, all female) of various TNM grades (tumor stage range: T1 to T4; node grade range: N0 to N2). Among these samples, 51 are infiltrating ductal carcinoma (IDC) and one is ductal carcinoma *in situ* (DCIS). As shown in Figure 3a, membrane-positive staining of RCA-I was observed in most of the TNBC patients (92%), showing slightly greater extents at later-stage cancers (100%) than earlier-stage cancer (87.1%).

In order to determine whether RCA-I binding exhibited any relation to the progression of TNBC in these tissue samples, we analyzed our findings with respect to both tumor stage and node grade. As shown in Figure 3b to e, the intensity and positive rates of RCA-I membrane staining indeed increased with each of these TNM grades. However, we note that the RCA-I staining intensities were better correlated with node grades than tumor stages (Figure 3e and f). It is known that late-stage cancer exhibits a higher metastatic potential compared to that of early-stage cancer, especially when the cancer is at a high node grade [31,32]. Thus, overall, these tissue microarray results are consistent with the lectin microarray results.

**Figure 4:**
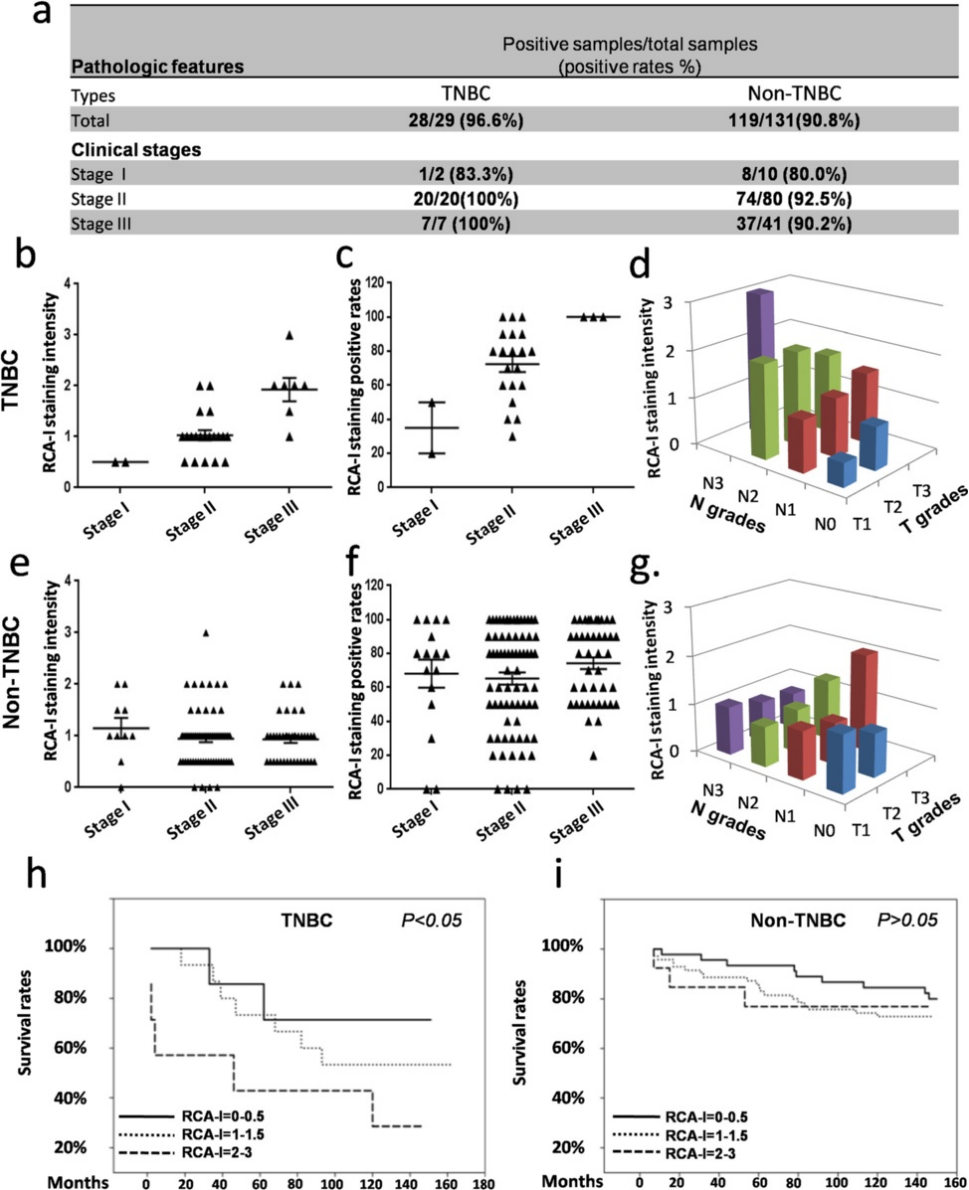
**Comparison of RCA-I bindings to clinical TNBC and non-TNBC samples. (a)** The summary of all clinical samples showed that there very high proportions of these samples were bound by RCA-I both in TNBC and non-TNBC samples. **(b and c)** The dependence of the mean intensities and positive rates of RCA-I membrane staining on the tumor stages in TNBC, whereas the independence of those in non-TNBC samples **(e and f). (d)** The dependence of the mean intensities of RCA-I staining on the T and N grades, whereas the independence of that in non-TNBC samples **(g). (h)** Kaplan-Meier survival curves on RCA-I staining intensities in TNBC and non-TNBC samples **(i)** showed RCA-I-binding intensities had the statistically significant power to stratify TNBC patients with distinct outcomes. RCA-I, *Ricinus communis* agglutinin I; TNBC, triple-negative breast cancer.

To further analyze whether the binding tendency of RCA-I is specific in TNBC samples and correlated with the prognosis of TNBC patients, another tissue microarray containing 160 clinical samples (median age 53.1 years, range 29 to 83 years, all female) of different TNM grades (tumor stage range: T1 to T3; node grade range: N0 to N3) from both TNBC (*n* = 29) and non-TNBC (*n* = 131) cancer patients with survival information was evaluated using IHC with RCA-I. As summarized in Figure 4a, membrane-positive staining of RCA-I was observed in the majority of both TNBC patients (96.6%) and non-TNBC patients (90.8%).

By comparing the intensities and positive rates of RCA-I membrane staining in TNBC and non-TNBC clinical samples, we found that the results from TNBC samples (Figure 4b and c) were in agreement with those from the aforementioned TNBC-only tissue microarray (Figure 3b and c). However, in non-TNBC samples, the intensities and positive rates of RCA-I membrane staining were quite similar across all clinical stages and did not show any specific proportionality (Figure 4e and f). Moreover, a similar correlation of RCA-I staining intensities with the node grades of the TNBC samples was also observed as in the TNBC-only tissue microarray (Figure 4d, and Figure S3, upper, in Additional file 3), whereas there was no such correlation in the non-TNBC samples (Figure 3g, and Figure S3, lower, in Additional file 3).

Using follow-up survival data from the 160 breast cancer patients, we stratified the breast-cancer-specific outcome according to the RCA-I membrane staining intensities. Scoring the RCA-I membrane staining intensities from 0 (low) to 3 (high), we found that indeed the patients could be stratified into good (RCA-I intensities ranged from 0 to 0.5), moderate (RCA-I intensities ranged from 1 to 1.5) and worse prognosis (RCA-I intensities ranged from 2 to 3) in terms of overall survival. The Kaplan-Meier curves showed a clear difference between these three groups (Figure 4h). A similar stratification was also evaluated with non-TNBC breast cancer patients, however no significant difference was observed with the Kaplan-Meier curves with this cohort (Figure 4i). Taken together, these results clearly indicate that RCA-I staining intensity shows a high correlation with the N grades of the TNBC samples, and also with the clinical outcome of TNBC patients, specifically.

### Inhibition of TNBC cell motility by RCA-I

**Figure 5:**
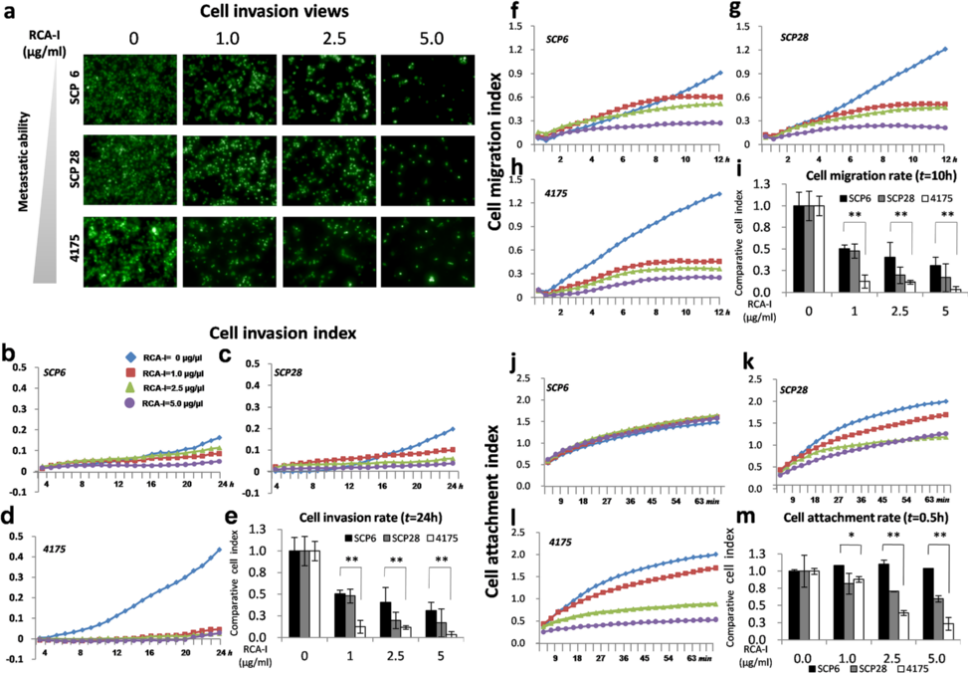
**Inhibition of TNBC cell motilities by RCA-I. (a)** Transwell assays were performed in the presence of different concentrations of RCA-I using three TNBC cell lines that differed in metastatic capacity. There is a clear dose-dependent inhibition of invasion by RCA-I with each cell line. **(b to m)** Cell motility characteristics determined with RTCA. In each figure, the concentration of RCA-I employed is indicated in the inset to Figure 4b. For the invasion, migration and adhesion assays, the cell index (CI) is linearly proportional to the number of invaded, migrated and adhered cells, respectively. **(b to e)** Invasion assays, **(f to i)** migration assays, and **(j to m)** adhesion assays. RCA-I, *Ricinus communis* agglutinin I; RTCA, real-time cell analyzer; TNBC, triple-negative breast cancer.

These results thus indicate that the presence of RCA-I-specific glycans on the cell surface, as judged by the extent of RCA-I binding, could be a useful indicator of metastatic potential in diagnostic applications. However, we also wondered whether the presence of these specific glycans might contribute to the development of this metastatic potential. We thus tested whether RCA-I binding *per se* affects any processes associated with metastasis. We first evaluated the effects of RCA-I on cell motility using matrigel-coated transwell assays. We studied three cell lines that were representative of high, middle, and low metastatic capacity (4175, SCP28 and SCP6, respectively). As shown in Figure 5a, the invasion capability of all three cell lines was inhibited by RCA-I, particularly at the highest RCA-I concentration. Significant differences among the three cell lines were, however, observed at lower concentrations of RCA-I. For example, the invasion rate of 4175 cells was sharply reduced at 1 μg/ml RCA-I while, at this RCA-I concentration, only a slight inhibition of the invasion rates of SCP6 and SCP28 cells was observed. This finding suggests that RCA-I-specific glycans may play a more significant role in the invasion capacity of those cells with the greatest metastatic potential. It should be noted that we also performed cell viability and aggregation assays with RCA-I to rule out the possibility that this lectin may induce aggregation and death of these TNBC cell lines, which may then contribute to the observed effects. However, as shown in Figure S4 in Additional file 4, the average cell volumes and viabilities of the cell lines incubated with RCA-I at the same concentrations as that used in the cell motility assays were not changed appreciably by the presence of RCA-I.

To obtain a more detailed and dynamic picture of this RCA-I-dependent inhibition of the motilities of these cell lines, similar experiments were performed using a RTCA that monitors the three key processes of tumor cell motility (namely, adhesion, migration, and invasion) in a real-time manner [21]. As shown in Figure 5b-d, the invasion rate of 4175 was greatly inhibited upon the addition of RCA-I at concentrations greater than 1.0 μg/ml, whereas those of SCP6 and SCP28 were only slightly inhibited compared to the corresponding controls in the absence of RCA-I (*P* <0.001). For example, after an incubation of 20 hr with 1.0 μg/ml RCA-I, the invasion rate of 4175 was 12.6% of the rate without added RCA-1, while those of SCP28 and SCP6 were 47.9% and 50.4%, respectively (Figure 5e).

As shown in Figure 5f-i, the results of the migration assay were similar to those of invasion assay, both in terms of the effects of RCA-I on different cells and the RCA-I concentration dependence. Interestingly however, the results from the adhesion assay were slightly different. In particular, as shown in Figure 5j, RCA-I did not alter the adhesion capability of SCP6 cells, while for SCP28 and 4175 cells, there was a clear reduction.

As a control, we also investigated the effects of another lectin, WFA, which exhibits similar binding extents to each of the SCP6, SCP28 and 4175 cells, in these TNBC cell motility assays. We found that the invasion capabilities of the cell lines were not changed by the presence of WFA (Figure S5 in Additional file 5). Hence, the aforementioned effects of RCA-I on TNBC cell motilities were indeed specific for RCA-I.

### Identification of RCA-I-recognized membrane glycoproteins

**Figure 6:**
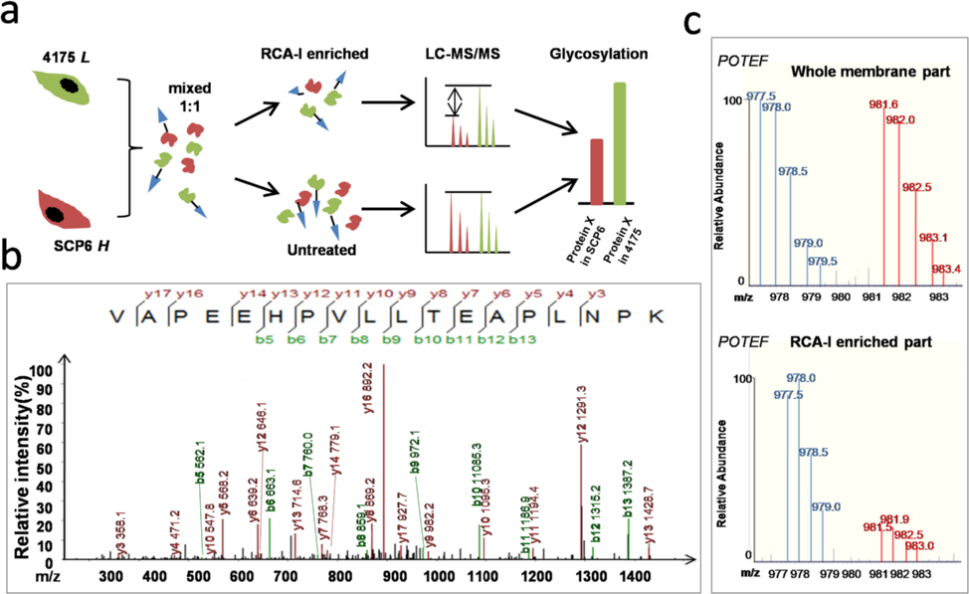
**The identification of RCA-I-recognized membrane glycoprotein by SILAC and LC-MS/MS analysis. (a)** A SILAC and LC-MS/MS combined strategy was used to detect both expression levels and galactosylations of the membrane glycoproteins captured by RCA-I. **(b)** LC-MS/MS spectrum of a peptide. The sequence of the peptide was identified as VAPEEHPVLLTEAPLNPK, which was from POTEF. **(c)** Representative base peak chromatogram of POTEF in the whole-membrane fraction (top) and RCA-I-enriched membrane fraction (down). The blue lines represent peptides of POTEF from 4175 cells whereas the red ones represent those from SCP2 cells (^13^C-labeled). LC-MS/MS, liquid chromatography-mass spectrometry/tandem spectrometry; POTEF, POTE ankyrin domain family member F; RCA-I, *Ricinus communis* agglutinin I; SILAC, stable isotope labeling by amino acids in cell culture.

In order to identify potential RCA-I-specific membrane glycoproteins in the TNBC cells, an integrated strategy combining SILAC and LC-MS/MS was employed (Figure 6a). Comparing the high/low (H/L) ratios of glycoproteins in the RCA-I-enriched fraction with the original whole-membrane protein fraction, both the changes of expression levels of target glycoproteins and their extents of galactosylation can be analyzed simultaneously.

As a result, a poorly characterized protein, prostate, ovary, testis-expressed POTEF, which belongs to the POTE membrane protein family, was identified in both the RCA-I-enriched part and the whole-membrane part (Figure 6b). It is noteworthy that the relative abundance of POTEF in the RCA-I-enriched fraction and the whole-membrane sample was 0.31 and 0.77, respectively (Figure 6c). Thus, the POTEF captured by RCA-I from the highly metastatic 4175 cells was about 2.5-fold greater than that from less metastatic SCP6 cells. Such a difference is likely owing to a greater extent of accessible galactose in POTEF in the 4175 cells.

## Discussion

Previous studies detailed distinct changes in glycan composition associated with the development of both tumors and metastases [33]. We reasoned that altered cell surface glycosylation might thus have potential in cancer diagnosis, for which there is a great need particularly in TNBC. As such changes might be associated with both large-scale and more subtle changes in glycan composition, we developed a unique high-throughput lectin microarray to enable multiplex characterization of cell surface glycans that is capable of identifying a wide range of differences in glycan composition [14].

With this array, we identified clear differences in the lectin-binding patterns between TNBC cells that differed in metastatic capacity (Figure 1). Further, as judged by the order of the cells in the heat map (Figure 1e), the degree of correlation among these multiplex lectin-binding patterns essentially followed the metastatic capacity of the cells. That is, the cells with the most similar lectin-binding patterns were also most similar to each other in metastatic capacity. Thus, widespread, subtle changes in cell surface glycosylation accompany the progression of the metastatic capacity of these cells. While the functional implications of these changes remains to be further investigated, this observation raises the possibility to use such multiplex lectin-binding patterns in future diagnostic applications, and perhaps identify subtle changes in cell surface glycan composition prior to the manifestation of significant metastases in these patients.

Yet an additional benefit of this means of characterization is the ability to identify specific lectins whose extents of binding might also directly correlate with metastatic capacity. Indeed, we found that the extent of binding of a single lectin, RCA-I, alone, was proportional to the metastatic potential of these cells. This result was also confirmed with patient-derived tissue microarrays (Figures 3 and 4). Hence, RCA-I tissue staining alone could serve as a potential index to quickly assess the risk of metastasis of TNBC patients and also predict the outcome of TNBC patients.

However, somewhat surprising, we also found that RCA-I might also prove useful in future studies aimed at understanding basic steps in TNBC metastasis, since its binding specifically and significantly inhibited TNBC invasion in the cell mobility assays. It is known that metastasis, especially hematogenous metastasis, is triggered by cell-cell interactions between cancer cells and vascular endothelial cells, followed by migration across the vascular wall, invasion into target tissues, and finally formation of the secondary tumor [34]. We found that RCA-I specifically inhibited cell adhesion more significantly than invasion or migration, with marked inhibition observed within 1 hr. Previously, there has been a great deal of focus on the migration and invasion steps of breast cancer cells metastasis [35]. However, the initial adhesion events of cancer cells are also clearly important, as they lead to the activation of cell surface receptors, release of chemokines, and are possibly associated with the formation of permissive metastatic microenvironment [36]. Our results thus implicate RCA-I-specific glycans as playing a critical role in this important first step, and suggest that future studies with RCA-I could provide more detailed understanding of its underlying molecular mechanisms. Further, with such an inhibitive effect on TNBC metastasis, such studies will also enable greater exploration of whether RCA-I could also be developed into useful therapy.

We also identified POTEF as a binding partner of RCA-I, and further, that it exhibits significantly altered extents of galactosylation in high/low metastatic TNBC cells. Although there is presently little known about this protein, most members of the POTE family are believed to be located on the plasma membrane and co-localize with actin filaments [37]. As is well known, actin filaments play important roles in cell motility and the establishment and maintenance of cell junctions to the extracellular matrix (ECM). Hence, this connection with actin may be related to the effects of RCA-I in cell invasion. Further, this identification, together with the observed effects of RCA-I in cell adhesion, suggest that the enhanced galactosylation of POTEF may be a contributing factor in this early step of TNBC metastasis.

Finally, we note that the procedures established here to study metastasis-associated cell surface glycans in TNBC are general and could be easily adopted for the study of other types of tumors. Lectin microarrays are an emerging technology that derived from well-established protein microarray techniques, which are capable of providing glycome-wide analyses of cell surfaces for rapid comparative screenings or more in-depth characterizations of the individual lectin-binding characteristics [38,39]. We thus expect further application of this technology will enable a greater understanding of the role of altered glycosylation in carcinogenesis and in other altered cellular states.

## Conclusions

Using a high-density lectin microarray, we screened six TNBC cells of different metastatic abilities from a common progenitor and found several lectins that exhibited altered bindings to these cells. In particular, a single-terminal galactose-binding lectin RCA-I showed a positive correlation with the metastatic capacity of these TNBC cells. Furthermore, FCM and immunofluorescence experiments using the same lectins validated the observations from the lectin microarray. The correlation between the extent of RCA-I binding and TNBC progress was also confirmed by both TNBC-only and overall breast cancer patient-derived tissue microarrays. Notably, the Kaplan-Meier curves from these tissue arrays showed a high correlation between RCA-I staining and the outcomes of TNBC patients. Thus, RCA-I could serve as a diagnostic biomarker to predict the risk of TNBC metastasis. Importantly, we discovered, for the first time, that RCA-I could inhibit TNBC cell adhesion, migration, and invasion to a degree that correlated with their metastatic abilities. We speculate that RCA-I inhibited the motilities of TNBC cells by blocking the metastasis-associated surface glycans and, hence, RCA-I might also have therapeutic potential. Finally, we identified a single membrane glycoprotein, POTEF, in the RCA-I-enriched fraction of proteins that exhibited markedly enhanced galactosylation in highly metastatic TNBC cells. Further investigation of this poorly understood membrane protein is needed to more fully understand its role in TNBC metastasis.

## Additional files

Additional file 1: Figure S1. The summary of bindings of lectins with six TNBC cell lines The bindings of all 91 lectins on the microarray and six TNBC cells of different metastatic abilities are summarized and displayed by fluorescent signal intensities. Additional file 2: Figure S2. The verification of RCA-I binding to TNBC and non-TNBC cell lines. (a) FCM analysis of the positive rates of RCA-I binding to eight TNBC cells and two non-TNBC cells. (b) RTCA analysis shows the relative invasion abilities of five TNBC cells and two non-TNBC cells. Additional file 3: Figure S3. The representative pictures of IHC assays of TNBC and non-TNBC tissue samples using RCA-I TNBC (red frame) and non-TNBC (green frame) tissue samples stained by RCA-I were displayed according to different T and N grades. Additional file 4: Figure S4. Cell aggregation and viabilities of TNBC cells treated with RCA-I. (a) Average cell volumes of the TNBC cell lines 4175, SCP28 and SCP6 treated with RCA-I of different concentrations. (b) Cell index which reflexes cell viabilities of TNBC cell lines 4175, SCP28 and SCP6 incubated with RCA-I of 0, 1.0, 2.5, 5.0 μg/ml, were detected by RTCA system. Additional file 5: Figure S5. Cell invasions of TNBC cells with WFA of different concentrations The invasion rates of the TNBC cell lines 4175, SCP28 and SCP6 treated with lectin WFA of the same concentration as RCA-I.

## Abbreviations

BSA: bovine serum albumin
CFDA-SE: carboxyfluorescein diacetate succinimidyl ester
CI: cell index
DCIS: ductal carcinoma *in situ*
DMEM: Dulbecco’s modified Eagle’s medium
ECM: extracellular matrix
ER: estrogen receptor
FBS: fetal bovine serum
FCM: flow cytometry
HER2: human epidermal growth factor receptor 2
HRP: horseradish peroxidase
IDC: infiltrating ductal carcinoma
IHC: immunohistochemistry
LC-MS/MS: liquid chromatography-mass spectrometry/tandem spectrometry
POTEF: POTE ankyrin domain family member F
PBS: phosphate-buffered saline
PBST: PBS with 0.5% Tween-20
PgR: progesterone receptor
PFA: paraformaldehyde
RCA-I: *Ricinus communis* agglutinin I
RTCA: real-time cell analyzer
SILAC: stable isotope labeling by amino acids in cell culture
SNA-I: *Sambucus nigra* agglutinin I
TNBC: triple-negative breast cancer
WFA: *Wisteria floribunda* agglutinin
WGA: *Triticum vulgaris* agglutinin
