# Supplementary figures and images for: Lectin RCA-I specifically binds to metastasis-associated cell surface glycans in triple-negative breast cancer
Source: Breast Cancer Res. 2015 Mar 11;17(1):36. doi: 10.1186/s13058-015-0544-9 (PMC4384317; doi:10.1186/s13058-015-0544-9)

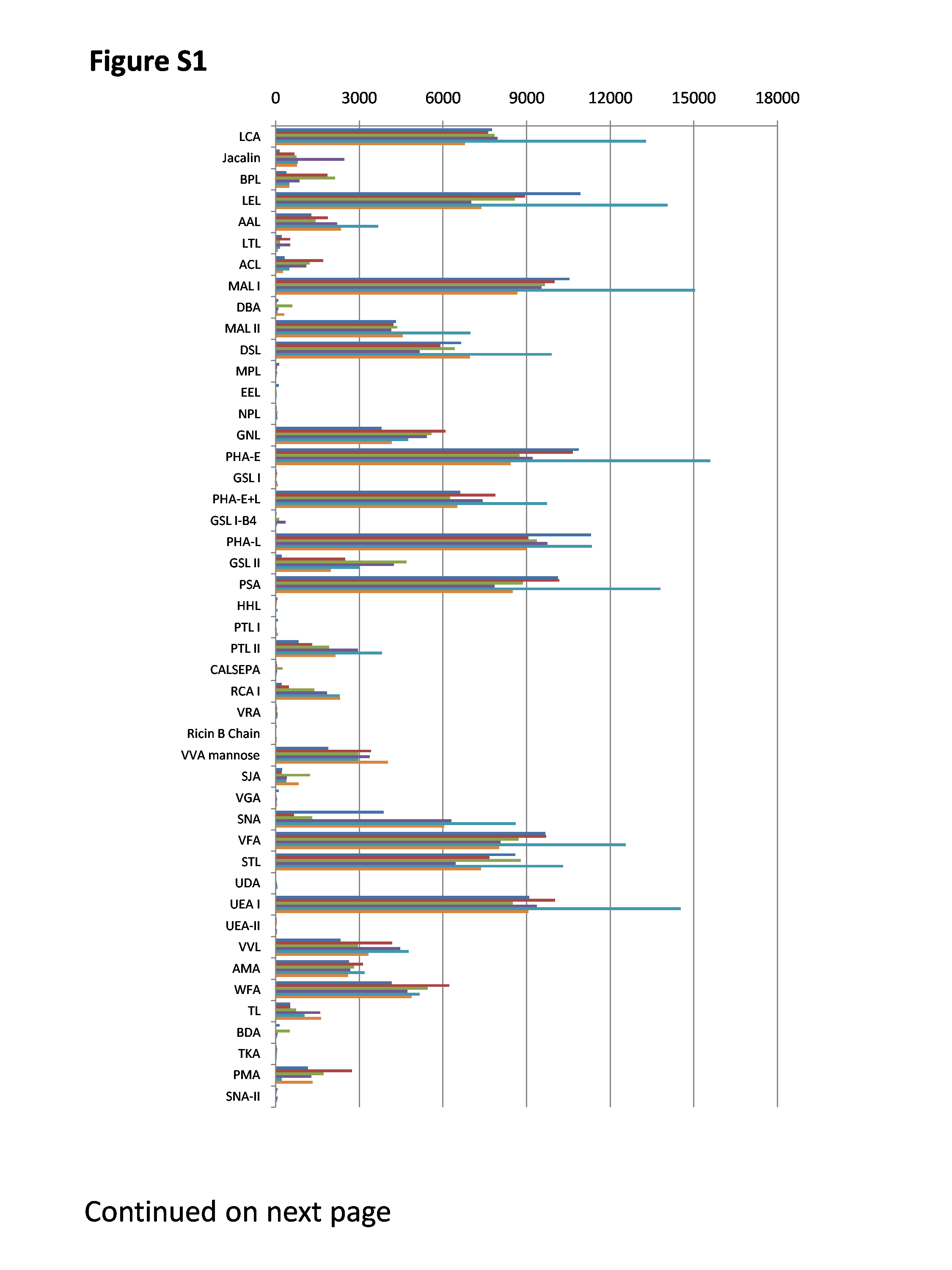

Supplement: Additional file 1: Figure S1. — The summary of bindings of lectins with six TNBC cell lines The bindings of all 91 lectins on the microarray and six TNBC cells of different metastatic abilities are summarized and displayed by fluorescent signal intensities. [file 13058_2015_544_MOESM1_ESM.zip › 1435877504129559_add1.tiff]

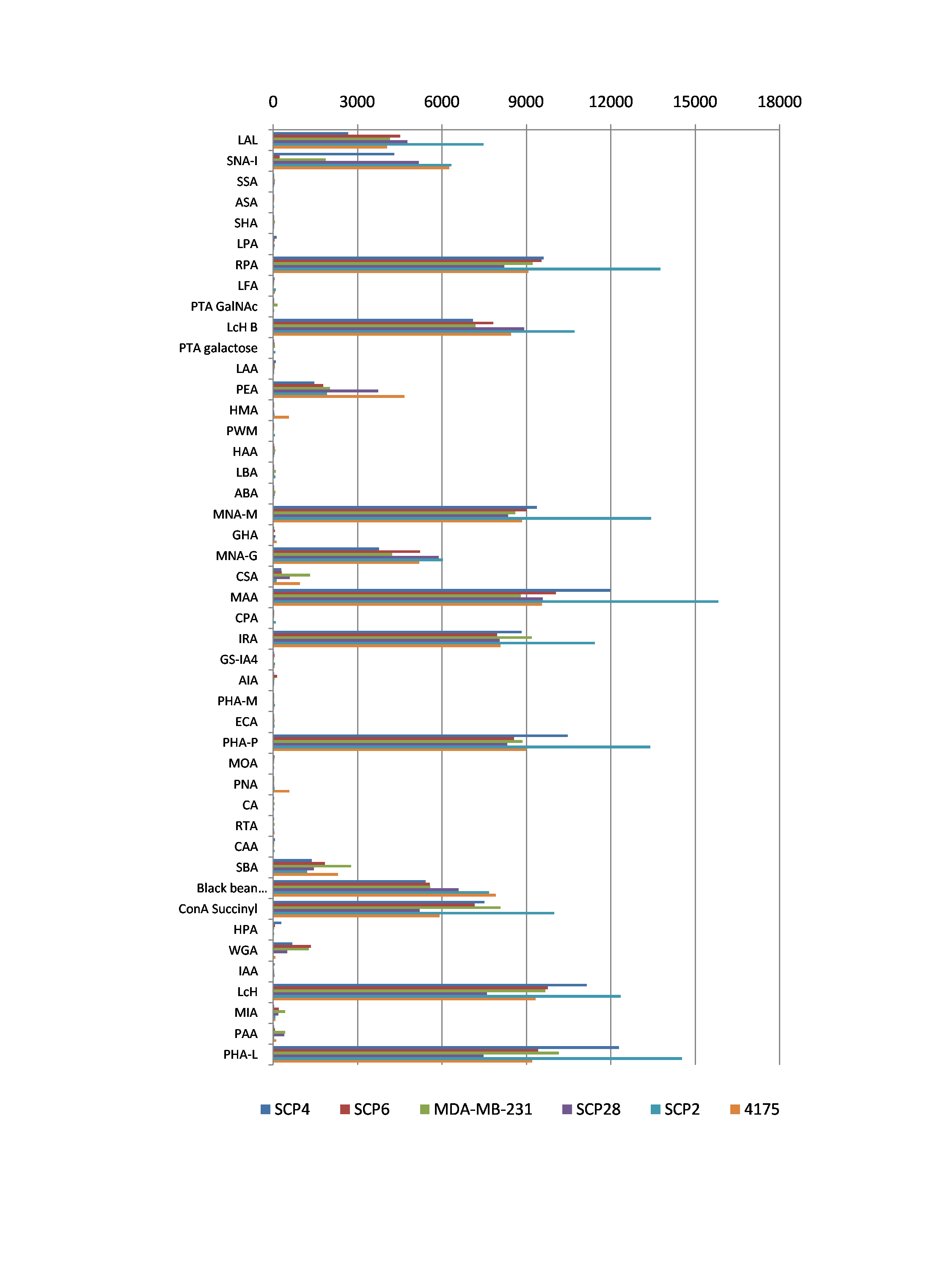

Supplement: Additional file 1: Figure S1. — The summary of bindings of lectins with six TNBC cell lines The bindings of all 91 lectins on the microarray and six TNBC cells of different metastatic abilities are summarized and displayed by fluorescent signal intensities. [file 13058_2015_544_MOESM1_ESM.zip › 1435877504129559_add2.tiff]

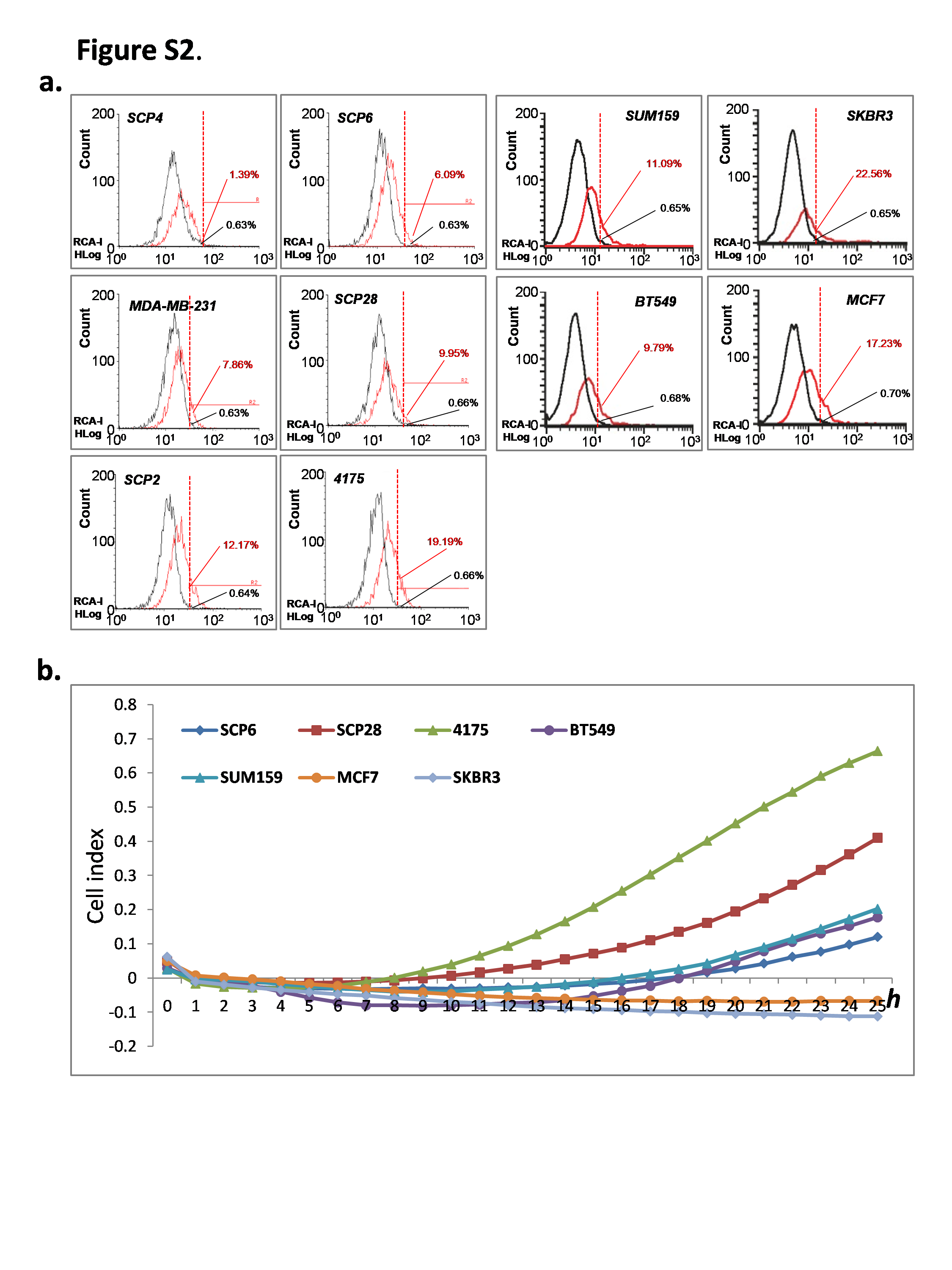

Supplement: Additional file 2: Figure S2. — The verification of RCA-I binding to TNBC and non-TNBC cell lines. (a) FCM analysis of the positive rates of RCA-I binding to eight TNBC cells and two non-TNBC cells. (b) RTCA analysis shows the relative invasion abilities of five TNBC cells and two non-TNBC cells. [file 13058_2015_544_MOESM2_ESM.tiff]

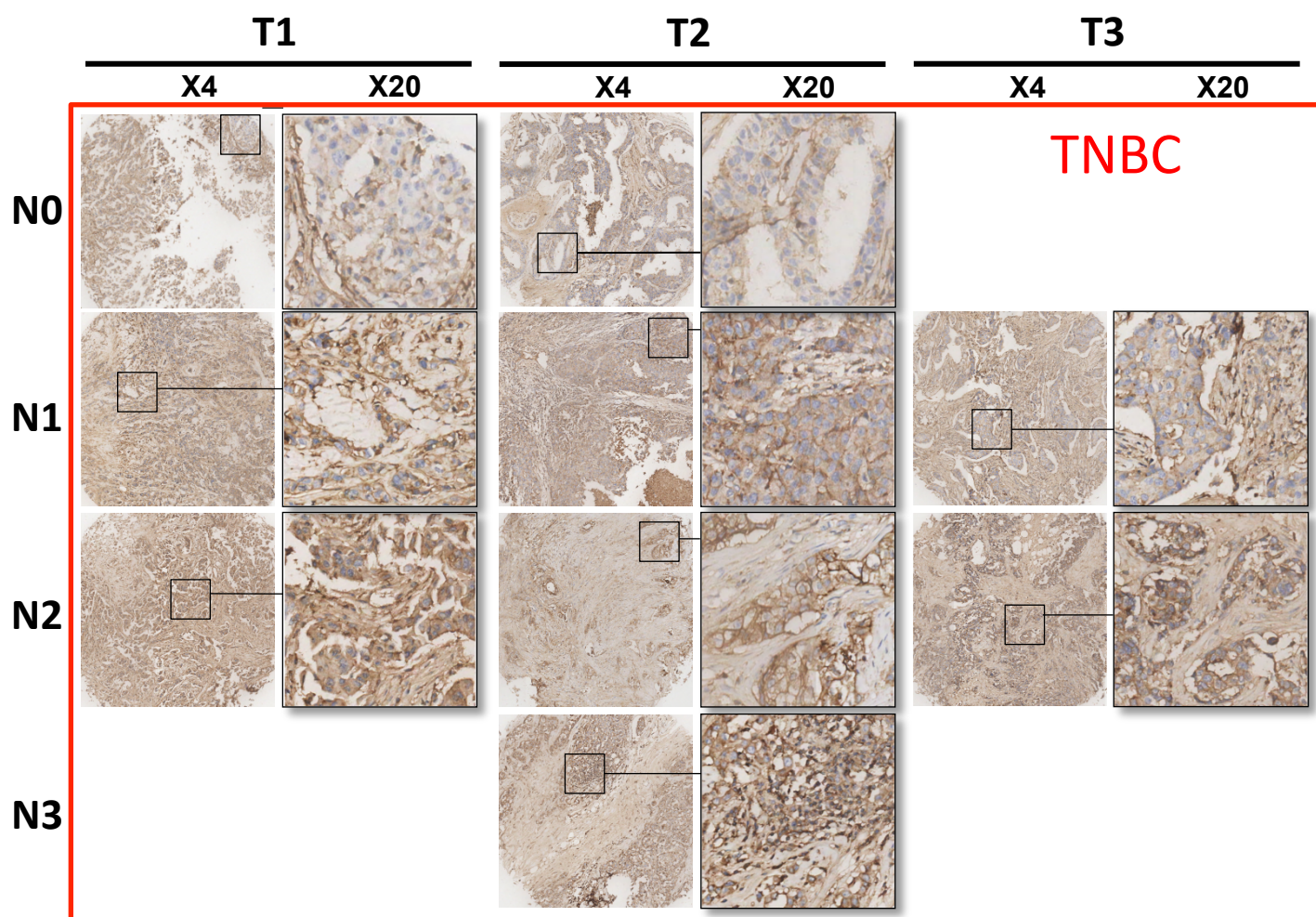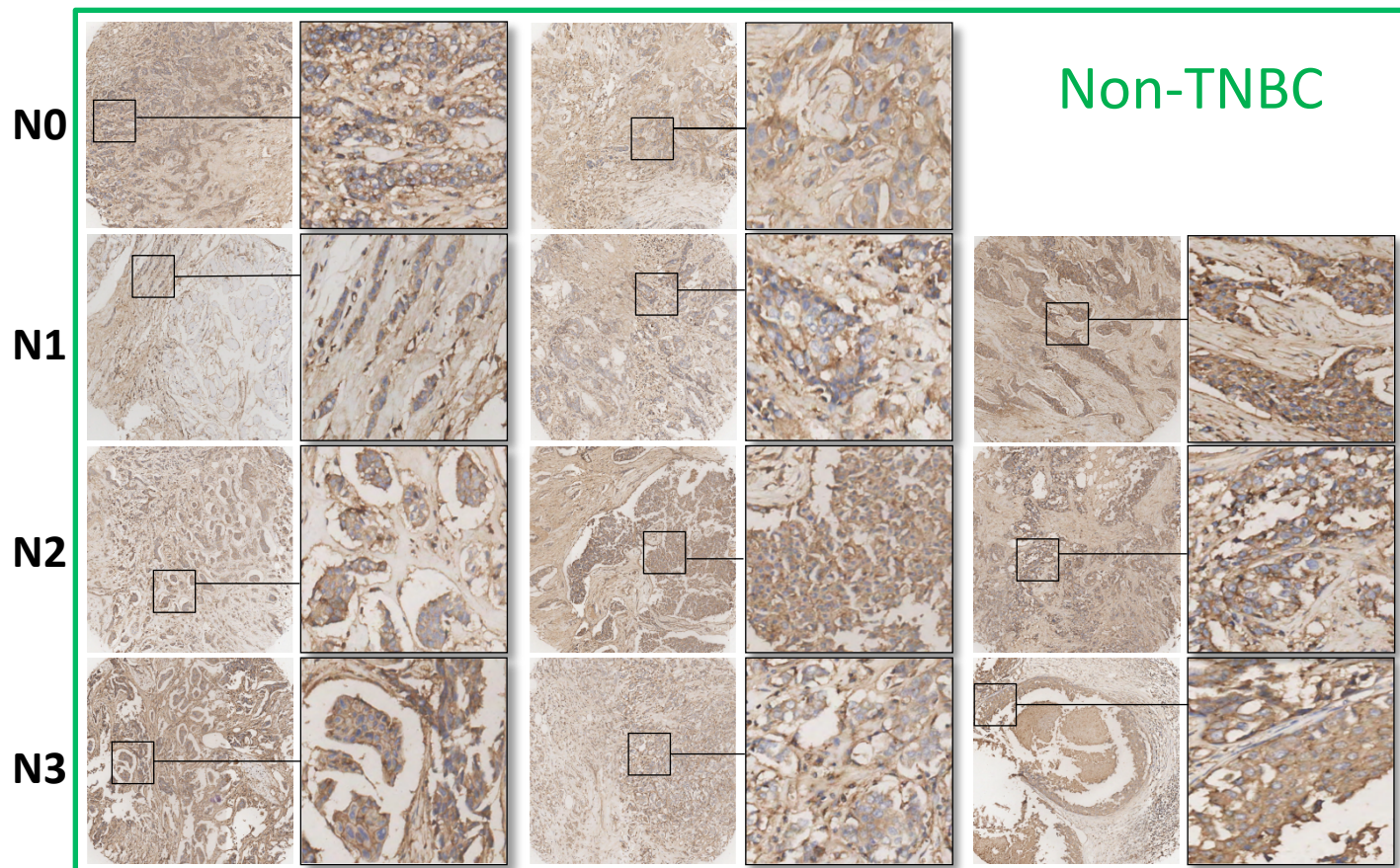

Supplement: Additional file 3: Figure S3. — The representative pictures of IHC assays of TNBC and non-TNBC tissue samples using RCA-I TNBC (red frame) and non-TNBC (green frame) tissue samples stained by RCA-I were displayed according to different T and N grades. [file 13058_2015_544_MOESM3_ESM.pdf]

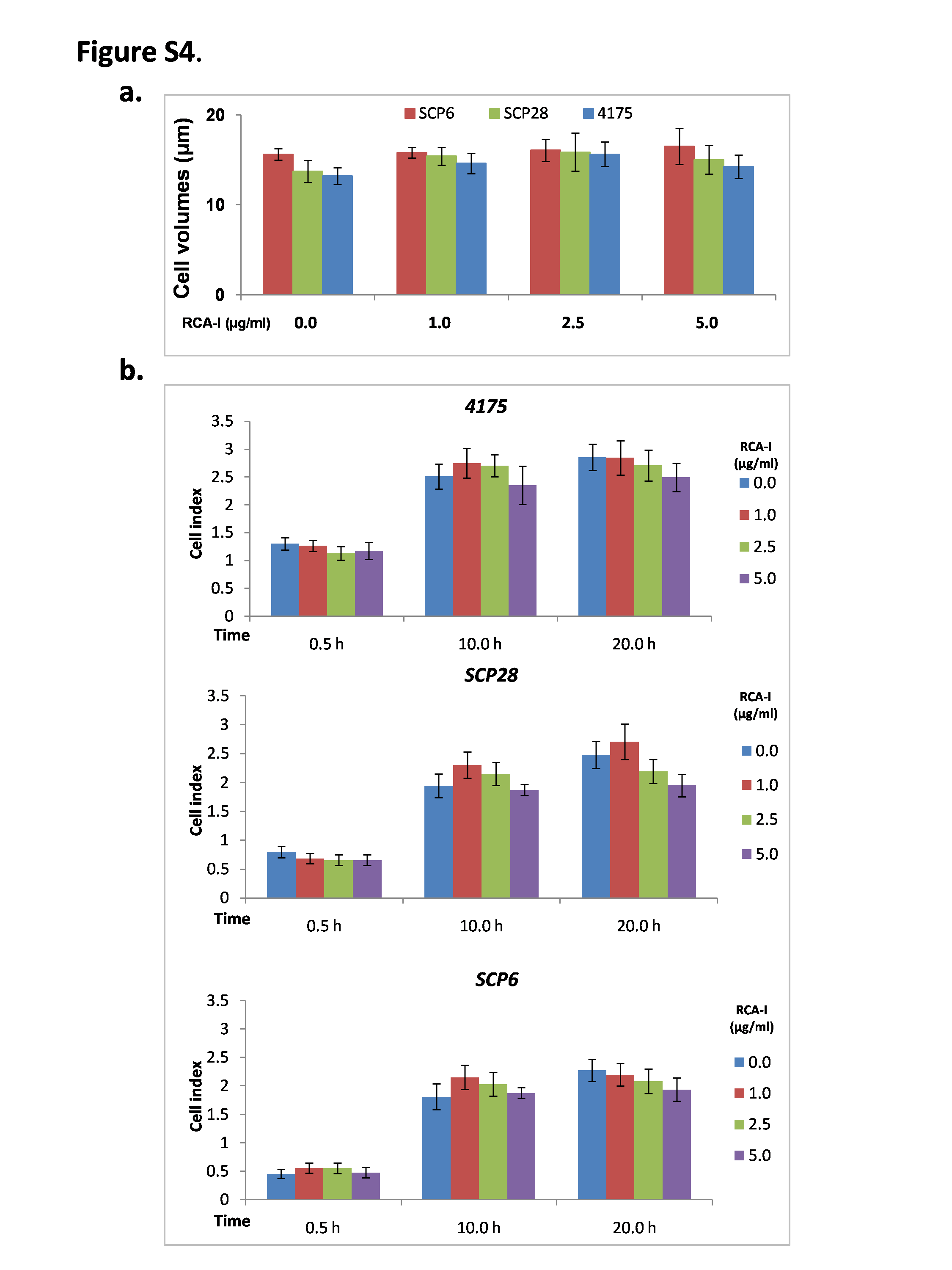

Supplement: Additional file 4: Figure S4. — Cell aggregation and viabilities of TNBC cells treated with RCA-I. (a) Average cell volumes of the TNBC cell lines 4175, SCP28 and SCP6 treated with RCA-I of different concentrations. (b) Cell index which reflexes cell viabilities of TNBC cell lines 4175, SCP28 and SCP6 incubated with RCA-I of 0, 1.0, 2.5, 5.0 μg/ml, were detected by RTCA system. [file 13058_2015_544_MOESM4_ESM.tiff]

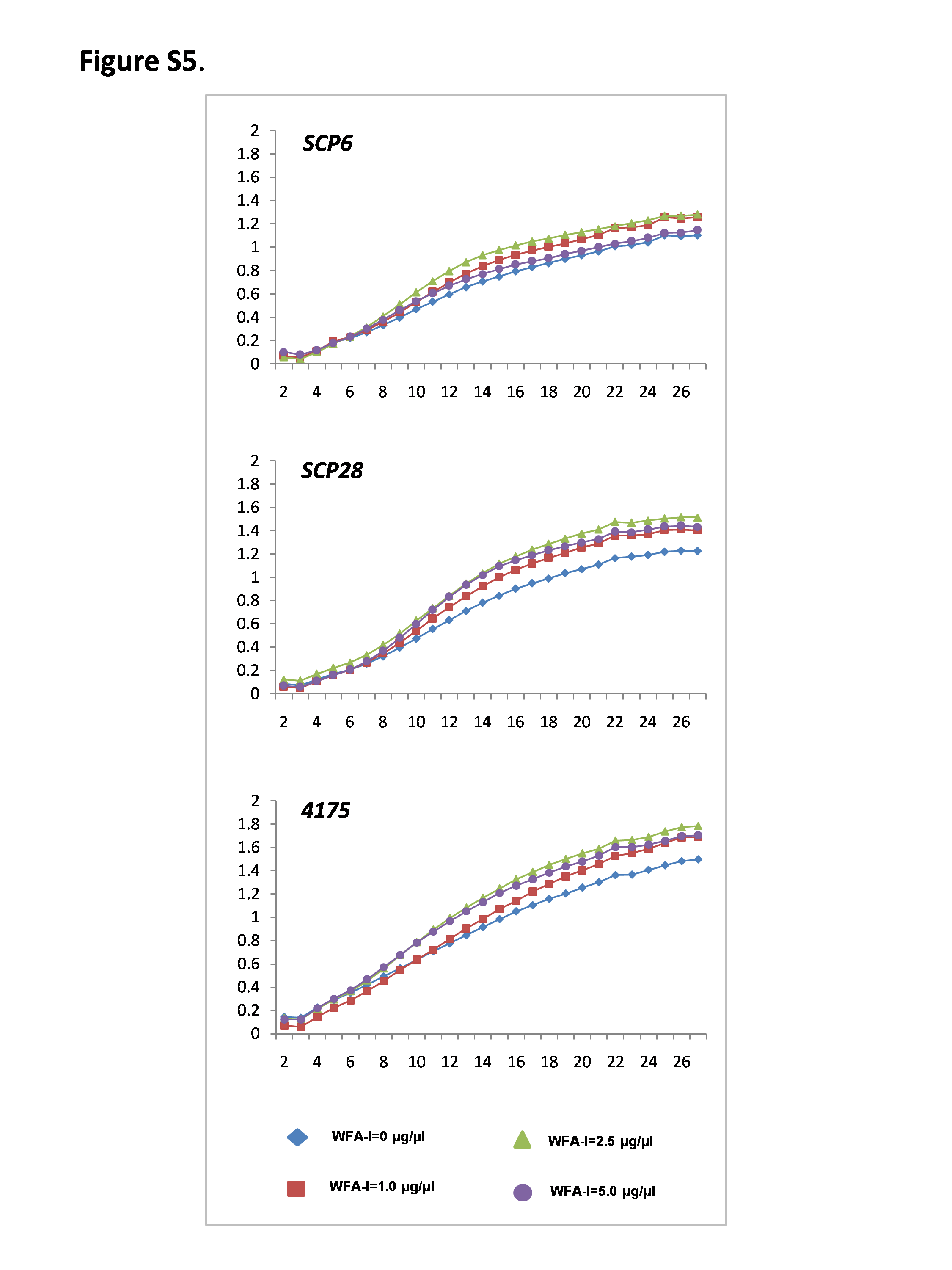

Supplement: Additional file 5: Figure S5. — Cell invasions of TNBC cells with WFA of different concentrations The invasion rates of the TNBC cell lines 4175, SCP28 and SCP6 treated with lectin WFA of the same concentration as RCA-I. [file 13058_2015_544_MOESM5_ESM.tiff]
